# Comparison among Different Green Extraction Methods of Polyphenolic Compounds from Exhausted Olive Oil Pomace and the Bioactivity of the Extracts

**DOI:** 10.3390/molecules29091935

**Published:** 2024-04-24

**Authors:** Valter F. R. Martins, Tânia B. Ribeiro, Ana I. Lopes, Manuela E. Pintado, Rui M. S. C. Morais, Alcina M. M. B. Morais

**Affiliations:** CBQF—Centro de Biotecnologia e Química Fina—Laboratório Associado, Escola Superior de Biotecnologia, Universidade Católica Portuguesa, Rua Diogo Botelho 1327, 4169-005 Porto, Portugal; s-vfrmartins@ucp.pt (V.F.R.M.); tribeiro@ucp.pt (T.B.R.); anlopes@ucp.pt (A.I.L.); mpintado@ucp.pt (M.E.P.); rcmorais@ucp.pt (R.M.S.C.M.)

**Keywords:** olive oil pomace, solvent extraction, ultrasound extraction, Ultra-Turrax extraction, cellulase, viscoenzyme, phenolic compounds, antioxidant activity, antimicrobial activity

## Abstract

The use of by-products as a source of bioactive compounds with economic added value is one of the objectives of a circular economy. The olive oil industry is a source of olive pomace as a by-product. The olive pomace used in the present study was the exhausted olive pomace, which is the by-product generated from the air drying and subsequent hexane extraction of residual oil from the olive pomace. The objective was to extract bioactive compounds remaining in this by-product. Various types of green extraction were used in the present study: solvent extraction (water and hydroalcoholic); ultrasound-assisted extraction; Ultra-Turrax-assisted extraction; and enzyme-assisted extraction (cellulase; viscoenzyme). The phenolic profile of each extract was determined using HPLC-DAD and the total phenolic content (TPC) and antioxidant activity (ABTS, DPPH, and ORAC) were determined as well. The results showed significant differences in the yield of extraction among the different methods used, with the enzyme-assisted, with or without ultrasound, extraction presenting the highest values. The ultrasound-assisted hydroethanolic extraction (USAHE) was the method that resulted in the highest content of the identified phenolic compounds: 2.021 ± 0.29 mg hydroxytyrosol/100 mg extract, 0.987 ± 0.09 mg tyrosol/100 mg extract, and 0.121 ± 0.005 mg catechol/100 mg extract. The conventional extraction with water at 50 °C produced the best results for TPC and antioxidant activity of the extracts. The extracts from the USAHE were able to inhibit Gram-positive bacteria, especially *Bacillus cereus*, showing 67.2% inhibition at 3% extract concentration.

## 1. Introduction

The plant *Olea europaea* L., commonly called the olive tree, is native to the Mediterranean region and occupies 11.6 million hectares globally [1]. This sector produces secondary, high-value by-products from which various beneficial chemicals, including dietary fiber, tannins, anthocyanins, polyphenols, and flavonoids, can be extracted. As a result, these by-products can be put to several uses, fulfilling the goals of the circular economy [1,2]. In actuality, 1 ha produces 2500 kg of olives annually, and 40–70 kg of olive pomace is produced for every 100 kg of olives [3]. In fact, 30 million m^3^ of olive mill waste, which is made up of olive wastewater and olive oil pomace, is produced annually worldwide because of the manufacture of olive oil [4]. A model of the economy employs residues as resources to be valorized, adhering to the 2030 Agenda of the United Nations under the heading of circular economy, which includes “zero waste production” [2]. These days, olive oil pomace is used in three different ways: as animal feed for cows, pigs, and chickens; in the energy industry; and directly in the soil as fertilizer [5,6,7,8]. Bioactive substances, such as hydroxytyrosol, maslinic acid, and oleanolic acid, are abundant in olive oil pomace. These substances exhibit favorable multifunctional characteristics, including anti-inflammatory, anti-microbial, antidiabetic, anticarcinogenic and anti-HIV effects [9]. According to many researchers, hydroxytyrosol possesses anti-inflammatory, anti-tumor, antiviral, antibacterial, and antifungal qualities [10,11]. According to Salucci et al. [12], tyrosol can strengthen intracellular antioxidant defenses. Because of its high hydrophilicity and low bioavailability, hydroxytyrosol is not easily incorporated into functional foods or pharmaceutical molecules, despite its impressive health benefits. This issue can be resolved with micro/nanoencapsulation [13,14]. After researching hydroxytyrosol and tyrosol from various olive extracts used in cosmetic creams, Miralles et al. [15] concluded that these compounds are intriguing ingredients in cosmetic products because of their antioxidant activity and stability under various temperature and light exposure conditions.

A number of studies, including Nunes et al. [16], have examined the content of olive pomace and discovered that it contains phenolic compounds, lipid acids, and vitamins, including MUFA and PUFA, and derivatives of hydroxytyrosol. There have been few studies carried out on deslipidified or exhausted olive oil pomace (EOP). Gómez-Cruz et al. [17] performed extractions of phenolic compounds from an EOP using different solvents: water, ethanol (20% and 50%) and water, and acetone and water. They determined the total phenolic content (TPC) and the antioxidant activity (ABTS, DPPH), performed HPLC analysis, and tested the antimicrobial activity of the extracts against *Listeria innocua, Staphylococcus aureus*, *Salmonella enterica* and *E. coli*. Gómez-Cruz et al. [18] performed ultrasound-assisted extraction with acetone (40%) and water solution to extract phenolic compounds from the same EOP, performed LC-MS and HPLC analysis, and determined the TPC and antioxidant activity of the extracts. Paz et al. [19] used a 50% hydroethanolic solution with 0.5% (*w*/*v*) of sulfuric acid to extract sugars, galacturonic acid, and phenols from another EOP. Olive pomace is typically rich in flavonoids like luteolin, quercetin, or rutin, phenolic acids like vanillin, caffeic acid, and coumaric acid, and secoiridoids and derivatives like oleoside and verbascoside [20]. It is also typically rich in phenolic compounds like hydroxytyrosol, tyrosol, and its derivatives.

The initial stage in separating bioactive natural compounds from raw ingredients is extraction. The extraction of bioactive chemicals from natural sources can be accomplished using various techniques. Although it takes considerable time and energy, solvent extraction does not require expensive equipment. The high-speed shearing homogenization equipment is called Ultra Turrax (UT). The material is subjected to extremely intense shear and thrust forces via the UT. For instance, it rapidly rotates blades to pulverize plant material while extracting bioactive chemicals using the appropriate extraction solvents [21]. Compared with UT, ultrasound offers a better extraction yield and requires shorter extraction periods. By breaking down the cell walls, this approach facilitates the contact of the solvent with the matrix [9]. Enzymes can cleave particular bonds of macro-elements, such as cell walls, and release bioactive chemicals by increasing their value and producing new derivatives in extracts, which makes extraction assisted by enzymes intriguing [22]. The enzyme cellulase, which is generated by fungi and bacteria, may hydrolyze vegetable cell walls by breaking down cellulose into glucose [23]. Viscoenzyme is a multicomponent carbohydrase that breaks down polysaccharides in plant cell walls through hydrolysis. This process cleaves the connections within the polysaccharide matrix, releasing components involved in intercellular communication [24].

The present study aims to make a comparison among several green methodologies to extract phenolic compounds from a Portuguese exhausted olive oil pomace (EOP). Several techniques, such as Ultra Turrax, ultrasounds, and the use of enzymes cellulase and viscoenzyme combined with more classical extraction methods, such as solvent extraction with water or a hydroalcoholic solution (90% ethanol), were tested. Chromatographic techniques, namely TLC and HPLC (standards of hydroxytyrosol, tyrosol, verbascoside, maslinic acid, coumaric acid, luteolin, vanillin, caffeic acid, rutin and catechol were tested), were used to identify and quantify the main phenolic compounds present in the EOP extracts. The yield of extraction was determined for each extraction condition, as well as the bioactivity of the extracts, through the antioxidant (ABTS, DPPH, and ORAC) and antimicrobial activities. The antimicrobial tests included *Yersinia enterocolitica*, *Bacillus cereus*, *Listeria monocytogenes, Staphylococcus aureus*, *Salmonella enterica* and *E. coli* bacteria. This work incorporates the concepts of “green chemistry” and “eco-extraction”, which aim to find extraction procedures that are high in extraction efficiency, while also being energy-efficient, user-safe, and ecologically benign.

## 2. Results and Discussion

### 2.1. Yield of Extraction

Nine different extraction conditions were used, performing macerations with hydroalcoholic solutions and water solutions, at 20 °C or 50 °C, and assisted by Ultra Turrax, ultrasounds, or enzymes, and the yield of extraction was calculated (Equation (1)) for each extraction. The results are presented in Table 1.

The results showed that the use of enzymes in extraction (experiments 6–9) allowed a higher yield of extraction. The cellulase degrades cellulose to glucose, hydrolyzing vegetable cell walls and releasing its content, and the viscoenzyme is involved in the cell wall disintegration of plant cell walls glycosidic bonds by hydrolysis, liberating more intercellular constituents [24]. These facts may explain the higher yield of extraction obtained using enzymes compared to hydroalcoholic solvents. However, no significant differences were found between the enzymatic and ultrasound-assisted enzymatic extractions using the same enzyme (experiment 7 compared to 6, and experiment 9 compared to 8). Wang et al. [25] presented a mechanistic approach where the application of ultrasound to the liquid extraction resulted in the enhancement of the phenolics yield of extraction. The different results of the present study may be due to the drying and the following hexane treatment applied to the olive pomace, which may have already changed the cell wall, making the combined use of enzymes and ultrasound no longer crucial in breaking them and releasing phenolic compounds. In fact, changes may occur in cellular structures, including cell membranes, during the hot-air drying process and this may also induce reactions, namely oxidation of the phenolic compounds [26]. Also, during the hexane treatment, non-polar bioactive compounds, such as alkaloids, aromatic hydrocarbons, terpenoids, coumarins and fatty acids, can be removed [27]. On the other hand, Stramarkou et al. [28] recovered polyphenols from olive pomace and observed a higher yield of extraction using methanol than water in conventional extraction, the same happening with the ultrasounds-assisted extractions. In the present study, the extraction with water presented a yield of extraction that was not significantly different than the one presented when using ethanol (experiments 5 and 1, respectively).

Table 2 provides a comparison of the advantages and disadvantages of each extraction method, helping in decision making for specific extraction needs.

During the preparation of the extracts, there were some challenges/limitations encountered: the dissolution of the extract was not always completed and there could be some precipitate that was filtered, and only the soluble compounds were analyzed; protection of the extracts from light always had to be performed, as phenolic compounds are sensitive to light; and the use of the ultrasound probe required an ice bath in order to avoid the increase in the temperature of the extraction solution containing an enzyme.

### 2.2. Thin Layer Chromatography (TLC)

**Figure 1:**
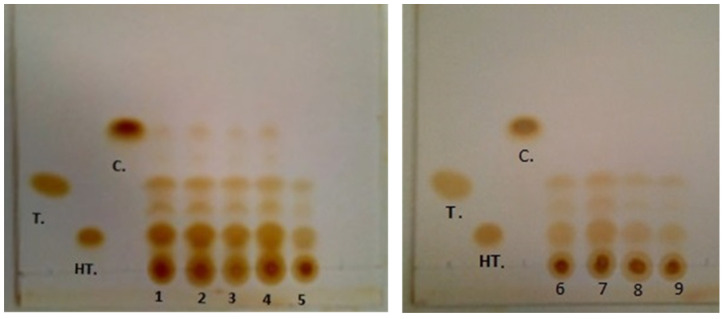
Thin layer chromatography (TLC) results. T—tyrosol; HT—hydroxytyrosol; C—catechol (standards); 1, 2, 3, 4, 5, 6, 7, 8, 9—samples; eluent used—toluene/acetone (9:1).

A TLC analysis was used to identify specific phenolic compounds in the exhausted olive pomace extracts and the results are presented in Figure 1 and Table 3.

TLC showed the presence of tyrosol and hydroxytyrosol in all analyzed samples, but catechol was present only after hydroalcoholic extraction (samples 1, 2, 3, 4), and this compound is absent in all aqueous extracts (samples 5, 6, 7, 8, 9). The values of RF were equal among samples of the same standard, for all standards, except for some samples where catechol was not present.

Capasso et al. [29] used the eluent solution benzene/ethyl acetate/methanol (60:30:10) and obtained the elution of the standard compounds in the same order, but with the RF values slightly different: 0.68 for catechol, 0.48 for tyrosol, and 0.26 for hydroxytyrosol.

### 2.3. High-Performance Liquid Chromatography and Diode-Array Detector (HPLC-DAD)

**Figure 2:**
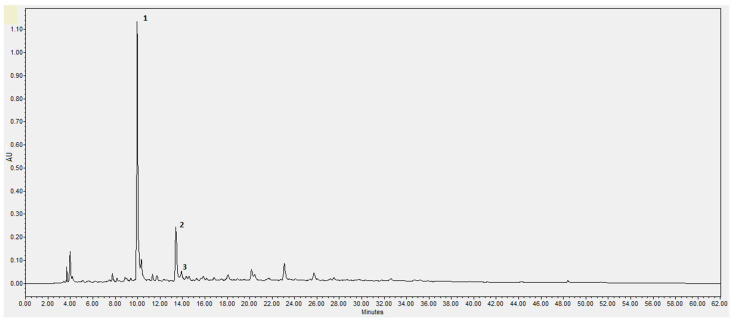
Example of a chromatogram from HPLC-DAD analysis of an extract from the hydroalcoholic extraction of the exhausted olive pomace. Peaks with high intensity identified: 1—hydroxytyrosol; 2—tyrosol; 3—catechol.

**Figure 3:**
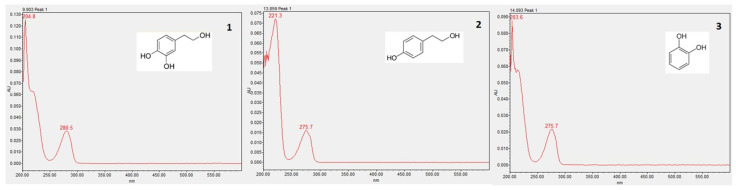
Absorption UV/Vis spectra of hydroxytyrosol (**1**), tyrosol (**2**), and catechol (**3**).

The phenolic compounds for each extract were quantified by HPLC-DAD. From all the standards used (hydroxytyrosol, tyrosol, verbascoside, maslinic acid, coumaric acid, luteolin, vanillin, caffeic acid, rutin and catechol), it was possible to identify only three compounds—hydroxytyrosol, tyrosol, and catechol—as it is possible to observe in the chromatogram (Figure 2). The absorption UV/Vis spectra of these three compounds are presented in Figure 3.

The LOD and LOQ values of hydroxytyrosol, tyrosol, and catechol are in Table 4.

The results in Table 5 were obtained in comparison with the standard compounds. The analysis was performed at 280 nm and the peak resolution level was performed. The linear equation for hydroxytyrosol was Y = 9 × 10^6^X + 2091.2 (R^2^ = 0.9967; X: 0.003125–0.8 mg/mL), with a retention time of 9.9 min and the compound maximum absorbance at 280.5 nm. The linear equation for tyrosol was Y = 6 × 10^6^X + 7481 (R^2^ = 0.9977; X: 0.003125–0.8 mg/mL) with a retention time of 13.8 min and the compound maximum absorbance at 275.7 nm. The linear equation for catechol was Y = 9 × 10^6^X − 9582.1 (R^2^ = 0.9999; X: 0.003125–0.2 mg/mL), with a retention time of 14.1 min and the compound maximum absorbance at 275.7 nm.

The main phenolic compounds found in all extracts were phenol alcohols, with the main compound being hydroxytyrosol followed by tyrosol (Table 5). Similar HPLC results were described by Gómez-Cruz et al. [17] about exhausted olive pomace extract obtained with water at optimal conditions (85 °C, 10% solids, 90 min). Gómez Cruz et al. [18] detected other minor phenolic compounds by capillary zone electrophoresis and LC-MS, including tyrosol hexoside, verbascoside, and luteolin and derivatives. In the present work, luteolin and verbascoside were not detected.

The extraction technique that used a hydroalcoholic solution and was assisted with ultrasounds (experiment 4) and Ultra Turrax (experiment 3) presented the highest contents in hydroxytyrosol. However, due to the yield of extraction, it was the methodology of water maceration assisted with the viscoenzyme (experiment 8) that presented the highest hydroxytyrosol content per gram of dry weight of the exhausted olive oil pomace. Similar results were obtained in relation to the other phenolic quantified compounds, tyrosol, and catechol, which presented the highest contents in extracts obtained by experiment 4 (Table 5). Gómez-Cruz et al. [17] also quantified hydroxytyrosol and tyrosol using HPLC and observed that hydroxytyrosol was the major phenolic compound in the aqueous extracts of EOP, as in the present study. Hydroxytyrosol in the extract was 6.74 mg/g DW EOP (extraction at 85 °C), while in the present study, it was lower, at 2.448 mg/g DW EOP, but the extraction was performed at a lower temperature (50 °C), or the EOP under study might be poorer in hydroxytyrosol. For catechol, the data agree with the results of the TCL analysis, which showed that catechol was present at low concentrations in hydroalcoholic extracts. However, this phenol was not detected by TLC analysis, whereas HPLC-DAD revealed a low concentration of catechol. Miklavčič Višnjevec et al. [30] quantified the phenolic compounds in olive pomace, which had been extracted with methanol–water and treated with hexane (delipidified), and obtained 157 ± 133 mg hydroxytyrosol derivatives/kg DW and 37 ± 30 mg tyrosol/kg DW. These values are substantially inferior to the ones of the present study, which may be due to the solvent used. Catechol was not identified. Niknam et al. [31] analyzed olive pomace, which had been pretreated with n-hexane, extracted with water/ethanol (50:50), and ultrasound-assisted and centrifuged, obtaining an extract with 14.51 ± 4.17 mg hydroxytyrosol/L and 4.58 ± 2.35 mg tyrosol/L.

With respect to the effect of the solvent used in the phenolics extraction, the quantification of these phenolics showed that the hydroalcoholic extraction (experiments 1–4) obtained higher contents of phenolics per gram of extract in comparison with the water extraction (experiments 5–9). Xie et al. [9] studied the effect of the content of ethanol in the extraction solution and obtained 56.16 mg hydroxytyrosol/g olive pomace after hydroalcoholic extraction, with 90% ethanol and 60.29 mg hydroxytyrosol/g olive pomace after hydroalcoholic extraction in 100% ethanol. These values are higher than the ones in the present study, but this study used exhausted olive oil pomace, which should be poorer in phenolic compounds.

The use of higher temperatures had no significant effect on the amounts of phenolics extracted by the hydroalcoholic extraction between 50 °C and 20 °C (experiments 1 and 2, respectively). On the other hand, the use of ultrasound (experiment 4) and Ultra Turrax (experiment 3), in general, increased the amounts of phenolics, hydroxytyrosol in particular. Xie et al. [9] compared the minimum temperature of 30 °C with 50 °C and obtained an increase of 50% in the yield of hydroxytyrosol. When the temperature was increased to 80 °C, a decrease in the phenolic concentration was observed, due to the phenolic compound degradation at a high temperature, as there was an overheating.

### 2.4. Total Phenolic Content (TPC) and Antioxidant Activity

The results on the TPC and antioxidant activity, namely ABTS (2,2′-azinobis(3-ethylbenzothiazoline-6-sulphonic acid), DPPH (2,2-difenil-1-picrilhidrazilo), and ORAC (oxygen radical absorbance capacity) of the extracts using the nine variations of the extraction parameters described in Table 1 are presented in Table 6.

The water maceration at 50 °C (experiment 5) was the extraction method that resulted in the highest TPC of the extract: 10.159 ± 0.741 (mg gallic acid equivalent/100 mg sample DW), and it also produced the best results of ABTS (69.155 ± 7.703 µmol of Trolox equivalent/100 mg sample DW), DPPH (38.12 ±1.614 µmol of Trolox equivalent/100 mg sample DW), and ORAC (215.522 ± 18.908 µmol of Trolox equivalent/100 mg sample DW). Gómez-Cruz et al. [17] also found that water extraction at 55 °C produced an extract with higher antioxidant activity (ABTS 70.7 ± 3.9/0.935 = 75.6 ± 4.2 mg TE/g DW EOP; DPPH 22.4 ± 0.82/0.935 = 23.96 ± 0.88 mg TE/g DW EOP; these values were calculated considering 6.5% EOP water content) than hydroethanolic extraction (water/ethanol 8:1, 5:5). Water extraction at 50 °C of EOP in the present study presented a lower ABTS 58.05 ± 6.47 mg TE/g DW EOP, but a similar DPPH 23.00 ± 0.97 mg TE/g DW EOP. TPC results (15.54 ± 1.13 mg GAE/g DW EOP) were lower in the present work than in Gómez-Cruz et al. [17] (40.7 ± 0.22 mg GAE/g DW EOP; this value was calculated considering 6.5% EOP water content). This may mean that the EOP of the present study was poorer in total phenolic compounds than the one of Gómez-Cruz et al. [17]. As far as we know, this is the first time that a Portuguese EOP has been studied. The other water solvent extractions assisted with enzymes or enzymes and ultrasounds (experiments 6 to 9) showed the lowest values of TPC and antioxidant activity. Gómez-Cruz et al. [32] found that lignin obtained by enzymatic hydrolysis of the EOP after aqueous extraction and pretreatment with 50% ethanol and 1% sulfuric acid showed lower TPC and antioxidant activity than the organosolv lignin from the pretreatment liquor. The results of the present study are in agreement with these findings. Ultra Turrax had a significant influence on the hydroalcoholic extractions (experiment 3 in relation to experiment 1), increasing the TPC, but had no positive effect on the antioxidant activity. Ultrasound, however, did not have a positive effect on this type of extraction (experiment 4 in relation to experiment 1), with respect to the same parameters. Gómez Cruz [18] found that hydroxytyrosol was the major phenolic compound (5.30 ± 0.03 mg/g DW EOP; this value was calculated considering 6.5% EOP water content) in the extract obtained using ultrasound-assisted extraction with acetone/water (4:6), for 12 min, temperature 26–46 °C. The content of this phenolic compound was lower (2.168 ± 0.307 mg/g DW EOP) in the present study, but an ethanolic solution was used. Increasing the temperature from 20 °C to 50 °C had a significant effect on the hydroethanolic extraction (experiment 1 in comparison with experiment 2), resulting in higher values of TPC and antioxidant activity (Table 6). Paz et al. [19] used a 50% hydroethanolic solution with 0.5% (*w*/*v*) of sulfuric acid at 97.95 °C for 23.18 min to extract sugars, galacturonic acid, and phenols of an EOP and found that the TPC was 20 mg/g DW EOP. In the present study, the TPC obtained in the hydroethanolic extraction at 50 °C was 8.916 ± 0.512 mg GAE/g DW EOP, which may be due to the lower temperature used since the authors state that the sulfuric acid is just a catalyst. Curiously, Stramarkou et al. [28] observed a decrease in TPC from 165.42 ± 4.74 to 89.6± 2.83 (mg GAE/g dry extract of olive pomace) when using ultrasound-assisted extraction in water at 25 °C in relation to the conventional extraction (without ultrasounds). And, for the same extraction conditions, these authors also observed a decrease in DPPH values. Miklavčič Višnjevec et al. [30] obtained a value of 414 ± 242 µg/mL for radical scavenging activity by DPPH EC50 for olive pomace extracts (ethanol/water, 80:20). Niknam et al. [31] analyzed a defatted (with hexane) olive pomace after ultrasound-assisted extraction with ethanol/water (5:5), similar to the exhausted olive pomace of the present study and he obtained TPC of 14.70 mg GAE/g and DPPH of 0.924 mmol Trolox/g, but the TPC value depended a lot on the ultrasonic homogenizer operating conditions. DPPH value is slightly higher than the result of the present study at 0.269 ± 0.023 mmol Trolox/g DW EOP. However, TPC was substantially higher at 82.03 ± 2.62 mg GAE/g DW EOP, which may be due to using a higher ethanol concentration (90%) in the extraction. Ribeiro et al. [20] analyzed crude olive pomace without deslipidification and they obtained 22.73–24.51 mg gallic acid equivalent (GAE)/g DW for TPC and 79.94–103.15 µM Trolox equivalent (TE)/g DW and 641.05–734.81 µM TE/g DW for antioxidant activity in DPPH and ORAC assays, respectively. Gómez-Cruz et al. [18], who also used an exhausted olive pomace (EOP; 6.5% water), obtained 47.69 ± 1.56 mg GAE/g DW EOP for TPC, and 135.91 ± 4.44 and 36.53 ± 0.353 mg TE/g DW EOP for ABTS and DPPH, respectively, after an acetone–water extraction assisted by ultrasounds (ultrasonic bath). The respective results of the present study were much lower than these findings—8.196 ± 0.262 mg GAE/g DW EOP and 37.296 ± 0.200 and 10.590 ± 0.912 mg TE/g DW EOP—but the extraction was performed with ethanol–water (experiment 4). Gómez-Cruz et al. [17] tested different solvents, water, ethanol 50%, and acetone 50% and obtained lower values, in general, than the former study [18].

### 2.5. Principal Components Analysis (PCA)

**Figure 4:**
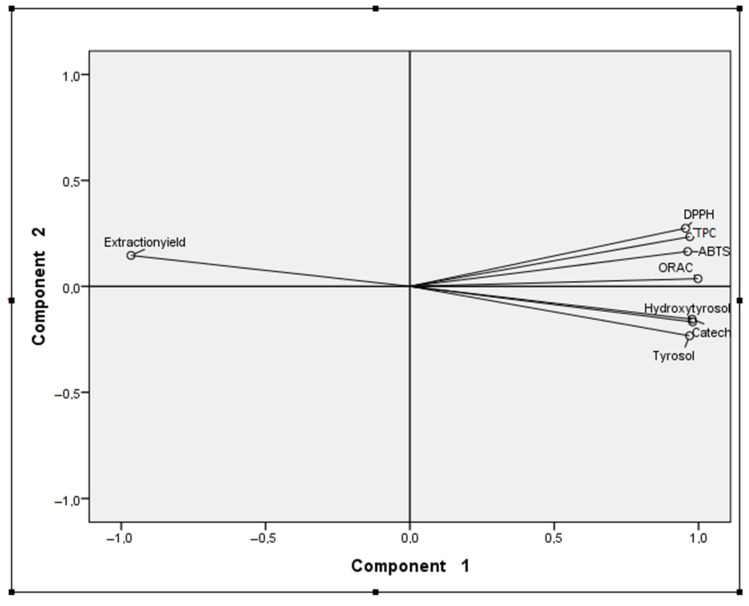
Principal component analysis (PCA)—projection of variables in the two first components.

**Figure 5:**
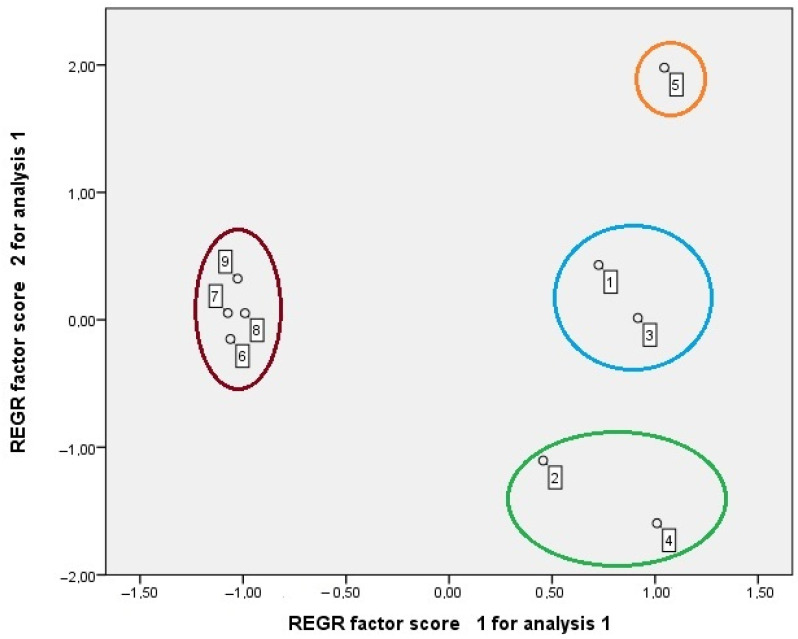
Principal component analysis (PCA)—scores of the extraction experiments in the sorting space built for the linear regression of the two first components.

The principal component analysis enables us to summarize, clarify, and simplify a group of information given by the variables. In this case, the different extraction experiments were used as independent variables. The yield of extraction, TPC, and antioxidant activity (ABTS, DPPH, and ORAC) and the concentrations of hydroxytyrosol, tyrosol, and catechol in the extracts were the dependent variables. Maintaining the original variation among extraction experiments, PCA allowed us to make a graphical representation of distance among the different extraction experiments and to identify similar groups or a better extraction methodology for one specific variable (Figure 4 and Figure 5).

In Figure 4, it is possible to observe the projection of variables and the two components explain a cumulative 98.10% of the total variance in the experimental data (component one is 94.52% and component two is only 3.58%). Component one is more correlated with all the variables than component two. The TPC, antioxidant activity, and concentrations in hydroxytyrosol, tyrosol, and catechol are positively correlated with component one and the yield extraction is negatively correlated with it. In relation to component two, the TPC, antioxidant activity, and yield of extraction are positively correlated with it, and the concentrations in hydroxytyrosol, tyrosol, and catechol are negatively correlated with it. The analysis of Figure 4 and Figure 5 allows us to observe four groups. Extraction experiment 5 presents a poor yield of extraction, a reasonable concentration of hydroxytyrosol, tyrosol, and catechol, and good TPC and antioxidant activity. The group with experiments 1 and 3 has a bad yield of extraction and medium values in the other variables. The group with experiments 2 and 4 has a poor yield of extraction, medium TPC, and antioxidant activity and presents good concentrations in hydroxytyrosol, tyrosol, and catechol (especially experiment 4). Finally, the group with extraction experiments 6, 7, 8 and 9 only has a good yield of extraction and bad results in the other variables.

PCA results allow us to evaluate and validate the differences among the extraction experiments on the yield of extraction, and antioxidant activity (AA), TPC, and phenolics concentrations, which were determined analytically, and the simple interpretation of the two graphs allow us to see what the behavior of each variable is (yield of extraction, concentration in phenolics, TPC and AA) for each extraction experiment. So, from Figure 4 and Figure 5, extraction experiments 6 to 9 revealed the highest yields of extraction, in agreement with Table 1; extraction experiment 5 showed the highest TPC and AA, supporting the results in Table 6; extractions experiments 2 and 4 presented the highest contents in hydroxytyrosol, tyrosol, and catechol, and, by analytical methods, experiment 4 was the one that presented the best results (Table 5).

### 2.6. Antimicrobial Analysis

The extract that showed the highest contents in the phenolic compounds hydroxytyrosol, tyrosol, and catechol, which was the one obtained by the ultrasound-assisted hydroethanolic extraction (experiment 4), was selected for the antimicrobial analysis.

**Figure 6:**
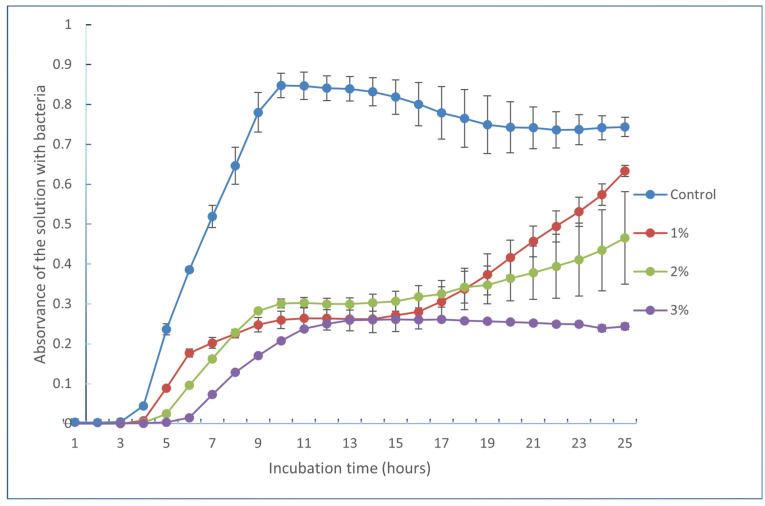
Inhibition curve of *Bacillus cereus* by the extract obtained from the ultrasounds-assisted hydroalcoholic extraction of exhausted olive oil pomace, at concentrations 1, 2, and 3%.

As an example, Figure 6 shows the inhibition curve for *Bacillus cereus* where it is possible to see the effect of a higher inhibition with the increase in the concentration, from 1% to 3%, of the extract obtained by hydroalcoholic maceration assisted by ultrasounds.

Table 7 summarizes the effect of the extracts obtained from the ultrasound-assisted hydroalcoholic extraction (experiment 4) on the growth inhibition of several bacteria. It is possible to observe that the extract from olive pomace was more effective in the inhibition of Gram-positive bacteria (*Staphylococcus aureus*, *Bacillus cereus*, and *Listeria monocytogenes)*. There is no effect or it is much lower on the Gram-negative bacteria (*Escherichia coli*, *Yersinia enterocolitica*, and *Salmonella enterica serovar enteriditis*).

According to the method used to evaluate the antimicrobial activity of the selected extract, the exhausted olive oil pomace (EOP) extract at 1, 2, and 3% was able to inhibit the growth of the tested bacteria, but there was a higher inhibition of the Gram-positive bacteria, especially *Bacillus cereus* and *Listeria monocytogenes,* and it increased with the increase in the concentration of the extract (Figure 6). Also, there was more than 50% inhibition of *Bacillus cereus* and *Listeria monocytogenes* with 3% extract from exhausted olive oil pomace. Ribeiro et al. [33] tested *Listeria monocytogenes, Bacillus cereus, Escherichia coli, Yersinia enterocolitica* and *Salmonella enteritidis* and the inhibition curve showed, in general, a retard in the lag phase, and the inhibition of the bacteria growth by extracts of olive oil pulp powder was not total. Gómez-Cruz et al. [17] tested the antimicrobial activity of aqueous EOP extracts, using a minimum inhibitory assay, and found that *L. innocua* and *S. aureus* (both Gram-positive bacteria) were the bacteria most susceptible to the bioactive agents of those extracts, while *E. coli* (MIC 45 mg/mL) and *Salmonella* sp. (MIC 40 mg/mL) (Gram-negative bacteria) were the most resistant. The present study presented similar results. Results observed on Gram-negative bacteria may be due to their membrane being rich in lipopolysaccharides, which restricts the penetration of foreign molecules [17]. Gómez-Cruz et al. [17] did not test *Yersinia* and *Bacillus cereus* bacteria.

## 3. Materials and Methods

### 3.1. Plant Material

The exhausted olive oil pomace was supplied in powder by Casa Alta—Sociedade Transformadora de Bagaços, a company from Ferreira do Alentejo, Beja, Portugal. It was obtained by processing olives harvested in 2021, in olive oil extraction units and after olive pomace drying and residual olive oil extraction with hexane. Therefore, lipophilic compounds, such as vitamin E and fatty acids, most probably have been removed during these processes.

The olive oil pomace was stored in hermetic containers in the dark and in dry air ambience (55% RH). The container was opened to weigh the samples just before use to avoid phenolic degradation.

### 3.2. Dry weight (DW) Determination

The dry weight of the exhausted olive oil pomace was determined by placing about 1 g of the sample in an oven (FP115, Binder, Tuttlingen, Germany) at 105 °C for 24 h [34]. The determinations were performed in triplicate. The dry weight was calculated through Equation (1).

Dry weight (%) = weight of dried sample (g)/weight of sample (g) × 100(1)

### 3.3. Extraction of Bioactive Compounds

To carry out the extraction, the best solvent/solute ratio was selected, as well as the temperature and combination of solvents and techniques, based on the literature [9,29]. Each extraction was performed in triplicate.

#### 3.3.1. Conventional Solvent Extraction

The classical solvent extraction (CSE) was performed with deionized water (A) or hydroalcoholic solution (B)—water/ethanol 1:9. An amount of 1 g of olive oil pomace was placed in 30 mL of the solution at 50 °C and 120 rpm (Orbital Shaker, MaxQ 6000, Thermo Scientific, Waltham, MA, USA) for 120 min (repeated twice). After that, the solutions were filtered, the ethanol was evaporated (Rotary Evaporator Buchi R-210, Buchi Labortechnik AG, Flawil, Switzerland) and lyophilized to obtain a dried extract (an extraction experiment was performed at room temperature, ca. 20 °C, as a control) [9].

#### 3.3.2. Ultra-Turrax-Assisted Extraction

The CSE was performed with a hydroethanolic solution (B) according to Section 3.3.1 and the mixture was homogenized with the Ultra Turrax (IKA T18 Digital Ultra Turrax, IKA, Staufen, Germany) for 2 min (pulses of 30 s). The solutions were filtered and lyophilized to obtain a dried extract.

#### 3.3.3. Ultrasound-Assisted Extraction (USAE)

The CSE was performed with a hydroethanolic solution (B) according to Section 3.3.1 and the mixture was homogenized with an ultrasound probe (Sonics, Vibra cell, Newtown, CT, USA), with 20 kHz pulses of 30 s for 10 min. The solutions were filtered and lyophilized to obtain a dried extract [9].

#### 3.3.4. Enzyme-Assisted Extraction (EAE)

Based on the CSE (A), olive oil pomace (1 g) was placed in 30 mL of water and the pH was adjusted to 5.0 prior to the addition of 1.0% of enzyme (cellulase or viscoenzyme; Celluclast, batch: CCN03196, Viscozyme L, batch: KTN02300, Novozymes, Bagsværd, Denmark). The extractions were performed at 50 °C and 120 rpm for 120 min (repeated twice). After, the mixtures were filtered and lyophilized to obtain a dried extract [35].

#### 3.3.5. USAE and Enzymes Extraction

The experimental processing conditions used were similar to EAE and USAE. Water (30 mL) was added with olive oil pomace (1 g) and the pH was adjusted to 5.0 prior to the addition of 1.0% of enzyme (cellulase or viscoenzyme L). The mixture was homogenized using an ultrasound probe (20 kHz pulses of 30 s for 10 min), maintaining the mixture in an ice bath. The solutions were filtered and lyophilized to obtain a dried extract.

#### 3.3.6. Yield of Extraction

The yield of extraction was calculated based on the amount of exhausted olive pomace (dry weight, DW) used in the extraction (Equation (2)).

Yield (g/g DW) = dried extract (g)/DW of exhausted olive oil pomace (g DW)(2)

**Figure 7:**
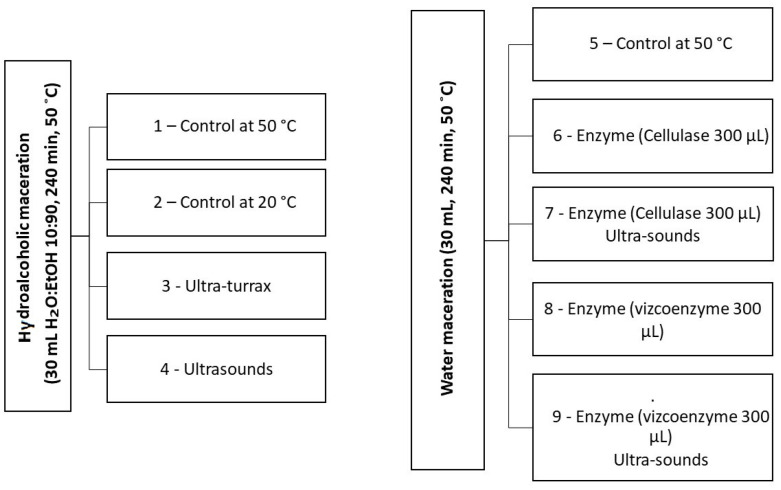
Performed extraction experiments.

The multiple techniques used to extract the polyphenols from the exhausted olive oil pomace are summarized in Figure 7.

### 3.4. Phenolic Compounds Identification and Quantification

#### 3.4.1. TLC Analysis

Thin layer chromatography (TLC) was performed in silica gel plates (Merck; Kieselgel 60; glace plates 10 × 10 cm; Darmstadt, Germany) according to Capasso et al. [29] with some modifications. The samples were eluted over a distance of 8 cm with eluent toluene/acetone (90/10%). The constituents were visualized using a revelation camera with iodine crystals, and by spraying with a 1% FeCl_3_ solution in water and heating for 10 min at 110 °C. The standards of hydroxytyrosol, tyrosol, and catechol (1 mg/mL) were used to identify the compounds. The extracts were dissolved in ultrapure water (50 mg/mL).

#### 3.4.2. HPLC-DAD Analysis

The main phenolic compounds identified were quantified using a HPLC-DAD (Waters Alliance e2695) with a separation module system interfaced with a photodiode array UV/Vis detector 2998 (PAD 190–600 nm) (Waters, Milford, CT, USA) according to Vilas-Boas et al. [36] with some modifications. The separation of the compounds was carried out in a reverse phase C18 column (Agilent Eclipse XDB-C18 5 µm 4.6 mm I.D. × 250 mm; Agilent, Santa Clara, CA, USA). The mobile phase was composed of solvent A—water/acetonitrile/TFA (94.8/5/0.2%)—and solvent B—acetonitrile/TFA (99.8/0.2%)—with the elution gradient at 0–1 min 0% B, 1–30 min 21% B, 30–42 min 27% B, 45–55 min 58% B and 55–60 min 0% B, and kept for 1 min at 0% B. The flow rate, oven temperature, and injection volume were 1 mL/min, 25 °C, and 20 µL, respectively. Detection was performed at 280 nm for hydroxybenzoic acid, 320 nm for hydroxycinnamic acid, and 360 nm for flavonols, and the data acquisition and analysis were carried out using Empower 3 Software.

Quantification was performed through calibration curves elaborated by using pure standards of hydroxytyrosol, tyrosol, verbascoside, maslinic acid, coumaric acid, luteolin, vanillin, caffeic acid, rutin and catechol. The samples and the dried extracts were dissolved in ultrapure water (50 mg/mL). Three independent analyses were performed in each of the triplicate extracts obtained in each extraction experiment.

### 3.5. Total Phenolic Content Determination

A CSE was performed with hydroalcoholic solution (water/ethanol 1:9). For this, exhausted olive oil pomace (1 g) was placed in 30 mL of the solution at 50 °C and 120 rpm (Orbital Shaker, MaxQ 6000, Thermo Scientific, USA) for 120 min (repeated twice). Then, the solution was filtered, the ethanol was evaporated, and the extract was suspended in water and lyophilized to obtain a dried extract. For the determination of the total phenolic content (TPC) and the antioxidant activity, 40 mg of this extract was suspended in 2 mL of distilled water (20 mg/mL). Three replicates were performed.

The TPC was determined by the Folin–Ciocalteu method described by Singleton et al. [37] and adapted for 96-well plate assay by Bobo-García et al. [38] with some modifications [36]. Shortly, the Folin–Ciocalteu reagent (100 µL, 20% (*v*/*v*) was added to the extract suspension (30 µL) and, then to sodium carbonate (100 µL, 7.4% *w*/*v*) and allowed to react in the dark at room temperature (ca. 25 °C) for 30 min. The absorbance was then measured at 765 nm (Synergy H1, Biotek, Winooski, VT, USA) in a 96-well microplate (Sarstedt, Numbrecht, Germany). Gallic acid was used as the standard for the calibration and the results were expressed as milligrams equivalent of gallic acid per 100 mg of dry weight extract (mg of GAE/100 mg DW). Three independent analyses were performed in each triplicate.

### 3.6. Antioxidant Activity Determination

The antioxidant activity of the extract solutions prepared above (20 mg/mL) was determined using three different methods.

#### 3.6.1. The ABTS Method

The ABTS (2,2′-azinobis(3-ethylbenzothiazoline-6-sulphonic acid)) assay was performed according to Gião et al. [39] with some modifications [40]. Shortly, the free radical ABTS was generated through a chemical oxidation reaction with potassium persulfate, with no involvement of an intermediary radical, and its concentration was adjusted with water to an initial absorbance of 0.700 ± 0.020 at 734 nm (Synergy H1, Biotek, Winooski, VT, USA). The extract suspension (20 µL) was allowed to react with 180 µL of the ABTS solution (2,2′-azinobis-(3-ethylbenzothiazoline-6-sulfonic acid) salt, 0.0384 g in 10 mL of ultrapure water mixed with a solution of potassium persulfate, 0.0066 g in 10 mL of ultrapure water) in the dark at room temperature (ca. 25 °C) and the absorbance was read 5 min exactly after in a 96-well microplate (Sarstedt, Numbrecht, Germany). The blank was distilled water (A0).

The inhibition percentage (I) of the sample was calculated using Equation (3). Trolox was used as the standard for the calibration and the results were expressed as µmol of Trolox equivalent/100 mg of extract dry weight (µmol TE/100 mg DW). Three independent analyses were performed in each triplicate.

I (%) = [(Abs A0 − Abs sample) ÷ Abs A0] × 100(3)

#### 3.6.2. The DPPH Method

The DPPH (2,2-diphenyl-1-picrylhydrazyl) assay was carried out according to the procedure described by Alexandre et al. [41] with some modifications [40]. Briefly, a stock solution (600 µM) was prepared by dissolving DPPH (23.6592 mg) in methanol (100 mL), and it was stored at −20 °C in the dark. The working solution (90 µM) was prepared by mixing 15 mL of the stock solution with 85 mL of methanol in order that the absorbance reached 0.600 ± 0.100 at 515 nm (Synergy H1, Biotek, Winooski, Vermont, USA). The extract suspension (25 µL) was allowed to react with the DPPH working solution (175 µL) in the dark at room temperature (25 °C) for 30 min in a 96-well microplate (Sarstedt, Numbrecht, Germany). The absorbance was then measured at 515 nm, with distilled water as the blank (A0).

The inhibition percentage (I) of the sample was calculated using Equation (3) and Trolox was used as a standard for the calibration. The results were expressed as µmol of Trolox equivalent/100 mg of extract dry weight (µmol TE/100 mg DW). Three independent analyses were performed in each triplicate.

#### 3.6.3. The Oxygen Radical Absorbance Capacity Method (ORAC)

The ORAC assay was performed in a black 96-well microplate (Thermo Scientific, Roskilde, Denmark), following the method described by Dávalos et al. [42] with some modifications [40]. The extract suspension (20 µL) was mixed with 120 µL of fluorescein (FL) solution (final concentration of 70 nM in the well) and 60 µL of AAPH (2,2′-azobis(2-amidinopropane) dihydrochloride), and the mixture was placed in each well. A control with 80 µL of 75 mM phosphate buffer (pH 7.4) and 120 µL of FL was used. A blank of FL and AAPH, using phosphate buffer in place of the antioxidant solution, was also used (Trolox). Eight calibration Trolox solutions (final concentration of 1–8 µM in the well) were used. The mixture was preincubated at 37 °C for 10 min. The AAPH solution (60 µL, final concentration of 12 mM in well) was added rapidly. After immediately placing the microplate in the reader, the fluorescence was recorded at intervals of 1 min for 90 min. A multidetector plate reader (Synergy H1, Biotek, Winooski, Vermont, USA) with 485 nm excitation and 528 nm emission filters was used. The equipment was controlled by the Gen5 Biotek software version 3.04. AAPH and Trolox solutions were prepared daily, and fluorescein was diluted from a stock solution (1.17 mM) in 75 mM phosphate buffer (pH 7.4). The antioxidant curves (fluorescence versus time) were normalized to the curve of the blank corresponding to the same assay by multiplying the original data by the factor fluorescence blank at t = 0 and dividing by fluorescence control at t = 0. The area under the fluorescence decay curve (AUC) was calculated from the normalized curves. The final AUC values were calculated by subtracting the AUC of the blank from all results.

The final ORAC-FL values were obtained using the standard curve and were expressed as µmol of Trolox equivalent/100 mg of dry weight extract (µmol TE/100 mg DW). Three independent analyses were performed in each triplicate.

### 3.7. Antimicrobial Analysis

The extract that showed the highest contents in hydroxytyrosol, tyrosol, and catechol, which was the one obtained by ultrasound-assisted hydroethanolic extraction, was selected for the microbial tests.

The antimicrobial assay was performed in a 96-well microplate (Thermo Scientific, Denmark), following the method described by Alexandre et al. [41] with some modifications [43]. An aqueous solution was prepared with the dried extract in a concentration of 1, 2, and 3% (*w*/*v*) [33]. Before the analysis, an overnight liquid culture of the selected bacteria was prepared in Mueller–Hinton broth (MHB) (Biokar Diagnostics, France), and the optical density was adjusted to 0.2 at λ = 610 nm, which corresponds to about 10^8^ CFU mL^−1^. Then, the liquid cultures were diluted in liquid culture to an inoculum concentration of 10^5^–10^6^ CFU mL^−1^. These liquid cultures and the extract solution (1, 2, and 3%) were transposed into a 96-well microplate (Sarstedt, Germany), and the optical density (OD) was registered at 600 nm for a 24 h period (1 h intervals) at 37 °C, using a microplate reader (Multiskan GO, Thermo Scientific, Vantaa, Finland). Inoculated MHB but without extract was used as the positive control, while MHB was used as the negative control. The increase in OD was considered to be a consequence of bacterial growth.

The inhibition percentage was calculated using Equation (4).

Inhibition (%) = ((OD_bacteria control_ − OD_bacteria_)/OD_bacteria control_) × 100 (4)

where OD_bacteria control_ and OD_bacteria_ represent the OD (at 600 nm) after 24 h of incubation of the control bacteria without and in the presence of the extract, respectively [36].

The biological material (bacteria) tested was from the CBQF collection and was obtained from rabbits and human clinical isolates. The species (Gram-negative) *Escherichia coli* ATCC 25922, *Yersinia enterocolitica* NCTC 10406, and *Salmonella enterica serovar enteriditis* ATCC 13076, and (Gram-positive) *Staphylococcus aureus* ATCC 6538, *Bacillus cereus* NCTC 2599, and *Listeria monocytogenes* NCTC 10357 were used.

### 3.8. Statistical Analysis

The data results were expressed as mean ± standard deviation of three independent extractions (*n* = 3). All statistical analysis was performed at a 5% significance level using SPSS Statistics software (IBM SPSS Statistics for Windows, Version 22.0 Armonk, NY, USA: IBM Corp.). Differences between means were analyzed using one-way analysis of variance (ANOVA). The ANOVA requirements, such as the normal distribution of the residuals and the homogeneity of variance, were tested by means of Shapiro–Wilk’s and Levene’s tests, respectively. All dependent variables were compared using Tukey’s post-hoc test. To summarize and analyze the data, a principal component analysis (PCA) was performed.

## 4. Conclusions

The present study delved into various green techniques for extracting bioactive compounds from an exhausted olive oil pomace, a natural source, with a particular focus on polyphenols renowned for their bioactivity and health-promoting properties.

Enzyme (cellulase; viscoenzyme)-assisted extraction, using ultrasound or not, exhibited superior yields in extraction efficiency, highlighting their potential for large-scale application. Additionally, ultrasound-assisted hydroalcoholic extraction at 50 °C (experiment 4) resulted in extracts with the highest concentration levels of hydroxytyrosol, tyrosol, and catechol, suggesting its efficacy in yielding potent bioactive extracts. Furthermore, the water extraction at 50 °C emerged as particularly promising, demonstrating high total phenolic content and antioxidant activity, indicative of its potential for various health and industrial applications. Moreover, taking into account the extract concentrations used (higher than 1%, 10 mg/mL), the extracts obtained in experiment 4 exhibited weak activity against *Bacillus cereus* and *Listeria monocytogenes*, with the potential to be used as adjuvants in packaging formulations of edible films and coatings, which are clean level formulations to decrease the growth of microorganisms.

From a circular economy perspective, the extraction of bioactive compounds from an exhausted olive oil pomace presents an environmentally sustainable approach, effectively repurposing waste material. The utilization of these bioactives as antioxidants and antimicrobials in food preservation or packaging (films and coatings), with potential health applications embodies a holistic approach towards sustainability.

In essence, the findings of the present study not only contribute to advancing various green extraction techniques, but also offer valuable insights into the diverse applications of bioactive compounds in promoting health and sustainability across different industries.

## Figures and Tables

**Table 1:** Yield of extraction (g/g DW) using different solvents and techniques.

Extraction Experiment	Yield of Extraction
1—50 °C H_2_O:EtOH	0.110 ± 0.02 ^b^
2—20 °C H_2_O:EtOH	0.083 ± 0.003 ^b^
3—Ultra Turrax + 50 °C H_2_O:EtOH	0.106 ± 0.02 ^b^
4—Ultrasounds + 50 °C H_2_O:EtOH	0.107 ± 0.004 ^b^
5—50 °C H_2_O	0.153 ± 0.002 ^b^
6—Enzyme (cellulase) + 50 °C H_2_O	0.512 ± 0.0006 ^a^
7—Ultrasounds + Enzyme (cellulase) + 50 °C H_2_O	0.455 ± 0.09 ^a^
8—Enzyme (viscoenzyme) + 50 °C H_2_O	0.493 ± 0.007 ^a^
9—Ultrasounds + Enzyme (viscoenzyme) + 50 °C H_2_O	0.492 ± 0.003 ^a^

Different letters mean significant differences between different extraction techniques (*p* < 0.05).

**Table 2:** Advantages and disadvantages of the different extraction methodologies used in this study.

Extraction Method	Advantages	Disadvantages
Water extraction	- Generally safe and environmentally friendly. - Simple and inexpensive. - Suitable for water-soluble compounds.	- Limited to polar compounds. - Ineffective for non-polar compounds.
Hydroethanolic extraction	- Versatile, extracts both polar and non-polar compounds. - Can be more efficient than water extraction for certain compounds. - Non-toxic solvent	- Extraction efficiency can vary. - Requires solvent recovery.
Ultrasound extraction	- Normally enhances extraction efficiency. - Reduces extraction time and solvent consumption. - Suitable for a wide range of compounds.	- Equipment can be expensive. - Energy intensive.
Ultra-Turrax extraction	- Relatively simple process. - Can be used at different scales.	- Requires significant energy.
Enzyme extraction	- Selective extraction. - Can be used under mild conditions.	- Enzymes can be expensive. - Extraction efficiency may vary with substrate and enzyme. - Requires the control of temperature and pH.

**Table 3:** Retention factor (RF) values.

Standards	Extracts Obtained in the Different Experiments								
	1	2	3	4	5	6	7	8	9
Hydroxytyrosol (0.13 ^a^)	0.12 ^a^	0.12 ^a^	0.12 ^a^	0.12 ^a^	0.12 ^a^	0.12 ^a^	0.12 ^a^	0.12 ^a^	0.12 ^a^
Tyrosol (0.30 ^b^)	0.31 ^b^	0.31 ^b^	0.31 ^b^	0.31 ^b^	0.29 ^b^	0.29 ^b^	0.29 ^b^	0.29 ^b^	0.29 ^b^
Catechol (0.51 ^c^)	0.51 ^c^	0.51 ^c^	0.51 ^c^	0.51 ^c^	0	0	0	0	0

Different letters in each row mean significant statistical differences (*p* < 0.05).

**Table 4:** LOD and LOQ values for hydroxytyrosol, tyrosol, and catechol.

Compound	LOD	LOQ
	(mg/100 mg extract)	
Hydroxytyrosol	0.002	0.005
Tyrosol	0.008	0.025
Catechol	0.007	0.021

**Table 5:** Phenolic compounds in the extracts of the exhausted olive pomace obtained by each extraction technique.

Extraction Experiment	Hydroxytyrosol (mg/100 mg Extract)	Hydroxytyrosol (mg/g DW Olive Oil Pomace)	Tyrosol (mg/100 mg Extract)	Tyrosol (mg/g DW Olive Oil Pomace)	Catechol (mg/100 Mgextract)	Catechol (mg/g DW Olive Oilpomace)	Total Phenolic Content (mg/100 mg Extract)	Total Phenolic Content (mg/g DW Olive Oil Pomace)
1	1.725 ± 0.115 ^b^	1.904 ± 0.127 ^e^	0.69 ± 0.052 ^b^	0.761 ± 0.057 ^d,e^	0.084 ± 0.006 ^c^	0.093 ± 0.006 ^e,f^	2.499 ± 0.162 ^c^	2.758 ± 0.179 ^e^
2	1.609 ± 0.103 ^b^	1.329 ± 0.085 ^f^	0.737 ± 0.095 ^b^	0.609 ± 0.078 ^e^	0.083 ± 0.009 ^c^	0.069 ± 0.007 ^f^	2.876 ± 0.092 ^b^	3.050 ± 0.097 ^d,e^
3	2.003 ± 0.087 ^a^	2.124 ± 0.091 ^d,e^	0.778 ± 0.046 ^b^	0.825 ± 0.048 ^c,d^	0.095 ± 0.012 ^b,c^	0.101 ± 0.013 ^d,e^	2.429 ± 0.176 ^c^	2.007 ± 0.146 ^f^
4	2.021 ± 0.287 ^a^	2.168 ± 0.307 ^c,d,e^	0.987 ± 0.094 ^a^	1.058 ± 0.101 ^b,c^	0.121 ± 0.005 ^a^	0.13 ± 0.005 ^a,b,c^	3.129 ± 0.341 ^a^	3.356 ± 0.366 ^b,c,d^
5	1.596 ± 0.135 ^b^	2.448 ± 0.207 ^a,b,c^	0.731 ± 0.066 ^b^	1.121 ± 0.101 ^a,b^	0.098 ± 0.015 ^b^	0.15 ± 0.023 ^a,b,c^	2.425 ± 0.209 ^c^	3.719 ± 0.320 ^a,b,c^
6	0.49 ± 0.080 ^c^	2.23 ± 0.368 ^b,c,d^	0.211 ± 0.047 ^c^	0.96 ± 0.211 ^b,c^	0.028 ± 0.004 ^d^	0.126 ± 0.017 ^b,c,d^	0.729 ± 0.129 ^d^	3.316 ± 0.588 ^a,b^
7	0.496 ± 0.028 ^c^	2.539 ± 0.143 ^a,b^	0.223 ± 0.026 ^c^	1.14 ± 0.131 ^a,b^	0.029 ± 0.003 ^d^	0.147 ± 0.015 ^a,b,c^	0.748 ± 0.054 ^d^	3.826 ± 0.276 ^a,b^
8	0.541 ± 0.031 ^c^	2.669 ± 0.151 ^a^	0.257 ± 0.031 ^c^	1.27 ± 0.156 ^a^	0.032 ± 0.005 ^d^	0.159 ± 0.026 ^a^	0.830 ± 0.063 ^d^	4.098 ± 0.304 ^a^
9	0.506 ± 0.043 ^c^	2.491 ± 0.212 ^a,b^	0.218 ± 0.028 ^c^	1.075 ± 0.138 ^b^	0.024 ± 0.007 ^d^	0.118 ± 0.036 ^c,d,e^	0.748 ± 0.064 ^d^	3.684 ± 0.313 ^a,b,c^

Different letters in each column mean significant differences (*p* < 0.05).

**Table 6:** Total phenolic content and antioxidant activity of nine different extracts of the exhausted olive oil pomace.

Extraction Experiment	TPC (mg Gallic Acid Equivalent/100 mg Dried Extract)	ABTS	DPPH	ORAC
		(µmol Trolox Equivalent/100 mg Dried Extract)		
1	8.116 ± 0.465 ^c^	58.421 ± 3.095 ^b^	30.458 ± 2.489 ^b^	187.625 ± 17.707 ^b,c^
2	6.813 ± 0.347 ^d^	37.415 ± 6.986 ^c^	25.408 ± 1.776 ^c^	170.896 ± 14.017 ^c^
3	8.743 ± 0.533 ^b^	57.152 ± 4.079 ^b^	30.873 ± 1.322 ^b^	205.297 ± 4.122 ^a,b^
4	7.666 ± 0.245 ^c^	63.528 ± 0.34 ^a,b^	25.099 ± 2.161 ^c^	201.182 ± 8.79 ^a,b^
5	10.159 ± 0.741 ^a^	69.155 ± 7.703 ^a^	38.121 ± 1.614 ^a^	215.522 ± 18.908 ^a^
6	3.057 ± 0.141 ^f^	18.760 ± 3.381 ^d^	10.601 ± 0.968 ^d^	69.336 ± 4.963 ^d^
7	3.658 ± 0.245 ^e,f^	18.665 ± 3.194 ^d^	9.873 ± 0.847 ^d^	66.555 ± 4.407 ^d^
8	3.73 ± 0.342 ^e^	24.103 ± 1.497 ^d^	10.439 ± 0.36 ^d^	63.979 ± 4835 ^d^
9	3.543 ± 0.293 ^e,f^	24.102 ± 1.497 ^d^	11.201 ± 0.755 ^d^	71.459 ± 6.156 ^d^

Different letters in each column mean significant differences between different extraction techniques (*p* < 0.05).

**Table 7:** Inhibition of bacteria (Escherichia coli, Yersinia enterocolitica, Salmonella enterica serovar enteriditis, Staphylococcus aureus, Bacillus cereus and Listeria monocytogenes) growth by the extract (concentrations 1, 2, and 3%) obtained through the ultrasounds-assisted hydroalcoholic extraction of exhausted olive oil pomace.

Bacteria	Inhibition (%) by Extracts from the Ultrasounds-Assisted Hydroalcoholic Extraction (Experiment 4)		
	1%	2%	3%
*Escherichia coli*	0.00 ^d^	7.65 ± 2.30 ^b^	5.20 ± 1.92 ^c^
*Yersinia enterocolitica*	0.72 ± 1.04 ^d^	7.93 ± 0.96 ^b^	10.98 ± 1.14 ^c^
*Salmonela enterica serovar enteriditis*	8.20 ± 0.54 ^c^	8.35 ± 0.71 ^b^	10.45 ± 0.59 ^c^
*Staphylococcus aureus*	2.78 ± 3.96 ^d^	18.68 ± 5.62 ^b^	42.67 ± 16.03 ^b^
*Bacillus cereus*	14.78 ± 1.19 ^b^	37.52 ± 16.5 ^a^	67.21 ± 1.97 ^a^
*Listeria monocytogenes*	20.79 ± 2.92 ^a^	36.45 ± 2.7 ^a^	59.83 ± 5.05 ^a^

Different letters in each column mean significant differences between different bacteria (*p* < 0.05).

## Data Availability

Data are contained within the article.
